# Interleukin-6 and pro inflammatory status in the breast tumor microenvironment

**DOI:** 10.1186/s12957-015-0529-2

**Published:** 2015-03-28

**Authors:** Alessandro Sanguinetti, Donatella Santini, Massimiliano Bonafè, Mario Taffurelli, Nicola Avenia

**Affiliations:** Struttura Complessa di Chirurgia Generale e Specialità Chirurgiche Azienda Ospedaliera ‘Santa Maria’, University of Perugia Azienda Ospedaliera Terni, Viale Tristano di Joannuccio, 105100 Terni, Italy; U.O. di Anatomia Patologica, Azienda Ospedaliera Policlinico S. Orsola-Malpighi, Bologna, Italy; Department of Experimental, Diagnostic and Specialty Medicine, Università di Bologna, Via Zamboni, 40126 Bologna, Italy; Dipartimento di Chirurgia generale e dei trapianti d’organo, Azienda Ospedaliera Policlinico S. Orsola-Malpighi, Via Pietro Albertoni, 40138 Bologna, Italy

**Keywords:** Interleukin-6, Pro inflammatory environment, Breast cancer stem cells

## Abstract

**Background:**

Greater than 50,000 new cases of breast cancer cases were diagnosed in Italy during 2013, with nearly 15,000 women succumbing to the disease. These epidemiological statistics highlight the overwhelming clinical dilemma of breast cancer and emphasize the need for novel therapeutic targets and prevention strategies. Countless studies in the fields of mammary gland development and breast cancer have led to an appreciation of a breast tumor microenvironment that actively contributes to the heterogeneous nature of breast cancer.

**Methods:**

The current review will focus on the impact of IL-6 and in the breast tumor microenvironment. Excessive IL-6 has been demonstrated in primary breast tumors and breast cancer patient sera and is associated with poor clinical outcomes in breast cancer. These clinical associations are corroborated by emerging preclinical data revealing that IL-6 is a potent growth factor and promotes an epithelial-mesenchyme (EMT) phenotype in breast cancer cells to indicate that IL-6 in the breast tumor microenvironment is clinically relevant.

**Results:**

High serum levels of interleukin-6 correlate with poor outcome in breast cancer patients. However, few data are yet available on the relationship between IL-6 and stem/progenitor cells, which may fuel the genesis of breast cancer *in vivo*. Mammospheres (MS) from node invasive breast carcinoma tissues express IL-6 mRNA at higher levels than MS from matched non-neoplastic mammary glands. IL-6 mRNA is detectable only in basal-like breast carcinoma tissues; our results reveal that IL-6 triggers a Notch-3-dependent upregulation of the Notch ligand Jagged-1, whose interaction with Notch-3 promotes the growth of MS and Michigan Cancer Foundation-7 (MCF-7)-derived spheroids. IL-6 induces a Notch-3-dependent upregulation of the carbonic anhydrase IX gene and promotes a hypoxia-resistant/invasive phenotype in MCF-7 cells and MS.

**Conclusions:**

In conclusion, our data support the hypothesis that IL-6 induces malignant features in Notch-3-expressing, stem/progenitor cells from human ductal breast carcinoma and normal mammary gland.

## Background

Interleukin 6 (IL-6), as major mediator of the inflammatory response, plays a primary role in the patho-physiology of cancer [1-4]. Cancer cells exposed to IL-6 or which secrete the cytokine as an autocrine factor, show malignant features, such as an enhanced capacity to invade the extracellular matrix and an increased drug resistance [5-7]. Based on these data, the inhibition of the IL-6/IL-6 receptor interaction with specific antibodies has been proposed as a support cancer therapy [8-10]. Further, high levels of *Notch* isoforms have been found to correlate with a poorer prognostic profile and reduced survival in breast cancer patients [11,12]. The molecular profile analysis of breast cancer stem tumorigenic cells revealed an upregulation of IL-6 and of Notch-3, a stem cell regulatory gene [13]. Stem/progenitor cells of the mammary gland reside in the basal cell layer and can be expanded *in vitro* from normal tissues as multi-cellular spheroids, named mammospheres (MS) [14]. MS regenerate and also form tubule-alveolar structures in Matrigel and in immunodeficient mice cleared of fat pads [15]. Similarly, MS from breast cancer tissues have been shown to proliferate *in vitro* and generate tubule-alveolar structures composed of CD44+/CD24− cells [16,17]. In this study, we try to evidence that IL-6 gene expression is upregulated in MS obtained from aggressive ductal breast carcinomas and that IL-6 regulates a Notch-3-dependent signaling pathway that promotes self-renewal and the invasive potentials of normal and tumor MS. *In vitro*, hypoxia actively maintains a stem cell/immature phenotype, induces a loss of differentiation markers, and blocks differentiation [18-20]. *In vivo*, stem cells express higher levels of hypoxia-regulated genes than the more mature progeny, as well as high levels of glycolytic enzymes [19,21]. In this study, we also try to evidence that IL-6 induced by the exposure to hypoxic stimuli, controls the expression of the stem cells regulatory gene Notch-3. Then, we report that an IL-6/Notch-3 interplay elicits an extracellular-signal-regulated kinase (ERK)-dependent upregulation of at least two genes: the Notch ligand Jagged-1 and the hypoxia survival gene carbonic anhydrase IX (CA-IX). We propose that the findings here reported may help in understanding the relationship among inflammation, hypoxia survival, cancer, and stem cells at molecular level.

## Methods

Ten fresh surgical specimens obtained from patients with ductal breast carcinoma, who underwent to quadrantectomy or mastectomy, were collected to generate MS. Normal and tumor samples were histologically characterized to ensure the proper classification of normal and tumor tissue. Immunohistochemistry was performed on formalin-fixed, paraffin-embedded tumor samples and on normal/tumor MS (N-/T-MS) (embedded in collagen (Sigma-Aldrich, St. Louis, MO, USA) 2 h before fixation in formalin). Tissues were histologically classified according to the WHO criteria and graded (G) following Elston and Ellis’ classification. The tumors were also typed by nuclear grading (NG) as follows: mild (NG1), moderate (NG2), and severe (NG3) nuclear atypia. Tumor size (pT) and axillary lymph node involvement (pN) were also recorded using pTNM (UICC) pathological staging criteria. Serial sections of formalin-fixed, paraffin-embedded samples were de-waxed, re-hydrated, and subjected to antigen-retrieval treatment. Tumor sections were stained using monoclonal antibodies anti-estrogen receptor (ER, clone 1D5), cytokeratin-5 (CK-5 clone D5/16B4), and epidermal growth factor receptor (EGF-R, clone DAHK1-WT) obtained from DakoCytomation (Glostrup, Denmark), ErbB-2 (HER-2, clone CB11), and cytokeratin-14 (CK-14, clone LL002) from BioGenex Laboratories (San Ramon, CA, USA), and CA-IX (M-75). Sections of normal and tumor MS were stained with anti CK-5, CK-14, EGF-R, CK-18 (CK-18, clone KSB17; Sigma-Aldrich, St. Louis, MO, USA), Oct-4 (clone c-20; Santa Cruz Biotechnology Inc., Santa Cruz, CA, USA), CD44 and CD24 (clone 156-3C11 and clone 24C02; Neomarkers, Portsmouth, NH, USA), CD133 (Miltenyi Biotec, Bergisch Gladbach, Germany), and E-cadherin (clone NCH38; DakoCytomation, Glostrup, Denmark). Antigens were unmasked with Tris-EDTA pH 9.0 at 98°C for 20 min, except for CA-IX antibody. Endogenous peroxidase activity was inhibited using a 0.5% H_2_O_2_ solution in methanol for 20 min, and sections were processed for immunohistochemistry with a non-biotin amplified method. Stained immunoreaction was quantified by image cytometry using Cytometrica software (C&V, Bologna, Italy). Sections were independently evaluated by two pathologists, and controversial results were discussed and defined. For ER immunostaining, the percentage of the labeled nuclear area over the total neoplastic nuclear area was assessed (<10% nuclei = negative, >10% nuclei = positive). A semi-quantitative assessment was applied for CK-5, CK-14, and EGF-R evaluation: cases were considered positive when the immunopositive neoplastic population was >10%. HER-2 staining was scored according to the HercepTest United States Food and Drug Administration-approved grading system. The percentage of immunopositive cells in normal and tumor MS was assessed on three to five sections (accounting from 100 to 300 cells as average). Total RNA was extracted from cultured cells, MS, and from archival tissues which had been frozen in liquid nitrogen at the time of surgical resection, using the RNA-extracting reagent TRIzol® (Invitrogen, Carlsbad, CA, USA). Primers used in the RT-PCRs are as follows: IL-6: annealing temperature 62°C, amplicon length 170 bp, F-5′-GAGAAAGGAGAC ATGTAACAAGAGT-3′, R-5′-GCGCAGAATGAGATGAGTTGT-3′; Notch-3: annealing temperature 62°C, amplicon length 93 bp, F-5′-TCAGGCTCTCACCCTTGG-3′, R-5′-AGTCACTGGCACGGTTGTAG-3′; CA-IX: annealing temperature 61°C, amplicon length 589 bp, F-5′-CAGGGACAAAGAAGGGGATGAC-3′, R-5′-TTGGAAGTAGCGGCTGAAGTCA-3′; Bmi-1, annealing temperature 62°C, amplicon length 220 bp, F-5′-GGAGACCAGCAAGTATTGTCCTTTTG-3′, R-5′-CATTGCTGGGCATCGTAAG-3′; Jagged-1: annealing temperature 62°C, amplicon length 170 bp, F-5′-TCGCTGTATCTGTCCACCTG-3′, R-5′-AGTCACTGGCACGGTTGTAG-3′; CK-5: annealing temperature 55°C, amplicon length 409 bp, F-5′-TAGGTGGTGGGCTCAGTGTGG-3′, R-5′-ACTTTGGGTTCTCGTGTCAGC-3′; CD133: annealing temperature 60°C, amplicon length 286 bp, F-5′-CTGGGGCTGCTGTTTATTATTCTG-3′, R-5′-ACGCCTTGTCCTTGGTAGTGTTG-3′; BCRP-I: annealing temperature 62°C, amplicon length 400 bp, F-5′-GTTTATCCGTGGTGTGTCTGG-3′, R-5′-CTGAGCTATAGAGGCCTGGG-3′; CD44: annealing temperature 62°C, amplicon length 300 bp, F-5′-CAGCAACCCTACTGATGATGACG-3′, R-5′-GCCAAGAGGGATGCCAAGATGA-3; Oct-4: annealing temperature 62°C, amplicon length 169 bp, F-5′-CTTGCTGCAGAAGTGGGTGGAGGAA-3′, R-5′-TGCCCGAAACCCACACTGCAG-3; Beta2 microglobulin: annealing temperature 58°C, amplicon length 180 bp, F-5′-ACCCCCACTGAAAAAGATGA-3′; R-5′-ATCTTCAAACCTCCATGA-3′. PCR primers and reagents were purchased from Invitrogen.

MS were obtained as described by Dontu *et al*., except that the methodology was downscaled to deal with low amounts of tissues 300 to 900 mg. Primary MS started forming after 4 to 6 days and were processed at day 10. Self-renewal of MS was tested by assessing the capacity of primary MS to generated secondary MS after trypsin disaggregation, as described by Dontu *et al*. Secondary MS were assessed at day 7. Cell invasion assay was performed on breast cancer cells (3 × 10^4^) and trypsin dis-aggregated MS and spheroids of Michigan Cancer Foundation-7 (MCF-7S) (5 × 10^2^ cells), as previously described. Six female BALB/c nude mice were injected with 5 × 10^5^ MCF-7 cells. Mice were followed up for 3 months, prior to being sacrificed. Three tumor xenografts were formalin-fixed, paraffin-embedded for immunohistochemical analysis or immediately frozen in liquid N_2_ for RT-PCR analysis. Cell death was induced by exposing MCF-7 cells, Hypo-7, and MS to DFX at a concentration of 100, 600, and 50 μM, respectively; MS were generated from the T-MS of the three patients with ductal breast carcinoma. It was found that T-MS were composed almost entirely by CD44+ (97% ± 3%), CD24− (<1%) cells, suggesting that the majority of cells in T-MS present a CD44+/CD24− cancer stem cell phenotype. IHC showed also that T-MS were composed by E-cadherin positive (97% ± 2%), CK-14 positive (99% ± 1%), and CK-18 positive (24% ± 7%) cells, revealing that T-MS are composed of epithelial cells showing ductal (CK-18) and luminal (CK-14) markers. RT-PCR analysis revealed that T-MS, but not the tumor tissues that T-MS had been obtained from, expressed detectable level of IL-6 mRNA. Compared to tumor tissues, RT-PCR analysis also revealed that T-MS expressed high levels of Bmi-1 mRNA, a gene associated with stem cell renewal, of CD44 mRNA, a gene whose expression has been associated with cancer stem cell phenotype in different organs. T-MS were then obtained from a set of samples (*n* = 10), in which also the normal mammary gland tissue was available to generate N-MS. Then, it assessed IL-6 mRNA in a set of archival breast tumor samples, including ductal (*n* = 6) and basal-like (*n* = 4) breast carcinomas, a subtype of cancer showing stem cell features. This tumor type, similar to MS, was characterized by the expression of CK-5, CK-14, EGF-R protein, as well as of Bmi-1 and CD133 mRNA, thereby reinforcing the notion of a tight similarity between MS and basal-like breast carcinoma cells. According to these results, we found that IL-6 mRNA was detectable in basal-like breast carcinoma tissues but not in ductal breast carcinoma. These results indicated that IL-6 expression occurs in MS obtained from aggressive ductal breast carcinoma and in basal-like breast carcinoma tissues, wherein stem cell-like phenotypes are particularly apparent. To test the functional role of IL-6 expression in MS, we exposed secondary T-MS to a monoclonal antibody that blocks the IL-6 receptor/ligand interaction (anti-IL-6, 1.5 μg/ml). Exposure of T-MS to anti-IL-6 antibody substantially blunted their secondary regeneration capacity, a functional property that has been referred to MS self-renewal capability. Likewise, we observed that, the administration of IL-6 (10 ng/ml) to N- and T-MS from the same patient yielded an increase in secondary MS formation compared to MS not exposed to the cytokine. We further investigated this phenomenon in the context of MCF-7-derived spheroids MCF-7S, which have been recently shown to contain a substantial proportion of CD44+/CD24− cells. MCF-7S expressed high levels of IL-6 mRNA, whereas the mRNA of the cytokine was absent in MCF-7 cells cultured in standard conditions. *Notch* genes play an essential role in MS self-renewal; in particular, Notch-3 is highly expressed in N-MS, and its blockage induces a marked reduction in MS self-renewal and survival. Notch-3 promotes MS survival and regulates the expression of its ligand Jagged-1; therefore, we next evaluated if Jagged-1 was involved in Notch-3-dependent MS growth. Indeed, either exposing N-MS to IL-6 (10 ng/ml) or adding anti-IL-6 (1.5 μg/ml) to T-MS modulated the expression of Jagged-1 mRNA. Moreover, we found that in MCF-7 cells, IL-6 elicited an upregulation of Jagged-1 mRNA, which was blocked by the co-administration of IL-6 with the MEK/ERK inhibitor UO-126; we, also, found that the upregulation of Jagged-1, induced by IL-6 was negligible in shN3/MCF-7 cells and that MCF-7S formation was extremely reduced when MCF-7 were transfected with a Jagged-1-specific siRNA (JAG1) compared to scrambled (SCR) control siRNA. ERK upregulation has been found to enhance the expression of the hypoxia survival gene *CA-IX*. Indeed adding IL-6 (10 ng/ml) to N-MS induced an upregulation of CA-IX mRNA. Increased CA-IX expression was also observed in MCF-7 cells exposed to IL-6 (10 ng/ml, 24 h), whereas CA-IX gene expression was markedly reduced by the administration of UO-126. This indicates that the CA-IX gene expression is regulated by IL-6/Notch-3 pathway in MCF-7 cells and MS. *CA-IX* gene, therefore, plays a crucial role in hypoxia survival of MS. These data indicate that IL-6/Notch-3-induced CA-IX gene expression promotes hypoxia survival in MS and supports the similarity between the gene expression profiles of MS and basal-like breast carcinoma tissues that suggested that the IL-6/Notch-3-dependent upregulation of CA-IX gene enhances the invasive behavior of MCF-7 cells and MS. Prompted by these observations, we assumed that IL-6 might regulate the production of its own mRNA. Consequently, we found that administration of IL-6 (10 ng/ml) upregulated IL-6 mRNA in MCF-7 cells and N-MS. Furthermore, once exposed to IL-6 (10 ng/ml for 24 h), MCF-7 cells expressed IL-6 mRNA, even 2 weeks after the withdrawal of IL-6 from the medium, suggesting that IL-6 auto-regulation might perpetuate phenotypic changes caused by exposing breast cancer cells to IL-6. Compared to untreated MCF-7 cells, such cells, referred to as MCF-7, revealed an upregulation of Notch-3 and CA-IX mRNA levels, paralleled by an enhancement in their invasive potential. The gene upregulation and the increase in invasive behavior of MCF-7 were abolished by the administration of anti-IL-6 (1.5 μg/ml), indicating that such features were dependent upon an autocrine IL-6 loop, and Notch-3 signaling was also required for this effect. These data support the argument that an IL-6 autocrine loop could induce a long-term enhancement in the aggressive features of breast cancer cells by sustaining an upregulation of the Notch-3/CA-IX axis.

## Results and discussion

In our study, we found that high IL-6 serum levels in breast cancer patients are associated with poor outcome and by the accumulating evidence suggesting that IL-6 assumes a direct role in the upregulation of malignant features in breast cancer cells. Herein, we have investigated the physiological effects and regulation of IL-6 in MS, which can be considered a suitable *in vitro* model for normal and tumor stem/progenitor cells of the mammary gland. In regard to the stem cell phenotype of MS, we here show that MS express a variety of genes which are upregulated in normal and cancer stem cell from various tissues, such as *Bmi-1*, *Oct-4*, *BCRP-I*, and *CD133*. Normal and tumor MS are almost entirely composed of CD44+/CD24− cells (the so-called breast cancer stem cell phenotype) and by a sub-population of cells (different in normal and tumor mammospheres from 10% to 20%) expressing CD133 protein. Moreover, normal and tumor MS express CK-5 gene which characterizes the basal cell compartment in which stem/progenitor cells of the mammary gland are harbored *in vivo.* T-MS obtained from node invasive tumors express higher IL-6 mRNA levels than MS obtained from normal tissue of the same patients. We also find that IL-6 mRNA levels are readily detected *ex vivo* only in CK-5 positive basal-like breast carcinoma tissues and how these tumors express high levels of stem regulatory gene *Bmi-1*. In addition, Bmi-1 is also upregulated in CD44+/CD24− breast cancer cells. We document that like basal-like carcinoma cells, T-MS also express the *CK-5/14*, *EGF-R*, *CD133*, *Bmi-1*, and *IL-6* genes. Thus, T-MS derived from ductal breast carcinoma would appear to possess at least some of the stem-cell-like characteristics of basal-like breast carcinoma that support the hypothesis that IL-6 gene expression is related to breast cancer stem cell phenotype [19]. We also demonstrate that the effects of IL-6 on MS require a functional Notch-3-signaling pathway. Notch-3, a member of the stem cell regulatory Notch family that governs stem cell homeostasis and turnover throughout species, modulates morphogenetic processes in the mammary gland; and when hyper-expressed in transgenic mice, Notch-3 also promotes mammary gland carcinogenesis. We also show that the CA-IX hypoxia survival gene is upregulated by IL-6 and also sustains the invasive potential of breast cancer cells and MS. In addition, CA-IX hyper-expression has been associated with reduced survival and poor outcome in breast cancer patients, and it has also been found to be over-expressed in basal-like breast carcinomas. Overall, the upregulation of *CA-IX* and *Jagged-1* adds to a growing number of genes, (that is, *CK-5*, *CK-14*, *EGF-R*, *CD133*, *CD44*, *Bmi-1*, *IL-6*) which also convey a tight similarity between T-MS and basal-like breast carcinoma. Finally, we show that IL-6 upregulates its own mRNA, thus perpetuating the effects of transient IL-6 exposure of breast cancer cells. In addition, this autocrine IL-6 loop requires active Notch-3 expression. In this investigation, we show that IL-6 triggers self-renewal and the invasive capacity of MS obtained from normal mammary tissue and that stem/progenitor cells are able to respond to an inflammatory stimulus (such as IL-6), by a process which promotes proliferation (and self-renewal) and stimulates the migration towards locations whereby tissue repair is required. In this regard, we examined the capacity of MS to generate three-dimensional structures that are to migrate into Matrigel and to generate multi-acinar and acinar/ductal structures.

## Conclusions

We provide evidence that T-MS obtained from node invasive tumors express higher IL-6 mRNA levels than MS obtained from normal tissue of the same patients. Conversely, no difference was found when MS from scarcely invasive tumors are examined. We also find that IL-6 mRNA levels are readily detected *ex vivo* only in CK-5 positive basal-like breast carcinoma tissues, an uncommon form of biological aggressive breast carcinoma with stem-cell-like features, including high levels of *CD133* and *CD44* expressions. We also show that these tumors express high levels of the stem cell regulatory gene *Bmi-1*, which was recently shown to be expressed at high levels in T-MS compared to their differentiated epithelial progeny. We also document that like basal-like carcinoma cells, T-MS also express the *CK-5/14*, *EGF-R*, *CD133*, *Bmi-1*, and *IL-6* genes. Thus, T-MS derived from ductal breast carcinoma would appear to possess at least some of the stem-cell-like characteristics of basal-like breast carcinoma. Our findings on basal-like breast carcinomas support the hypothesis that IL-6 gene expression is related to breast cancer stem cell phenotype. We also provide evidence that the effects of IL-6 on MS require a functional Notch-3-signaling pathway. Notch-3, a member of the stem cell regulatory Notch family that governs stem cell homeostasis and turnover throughout species, modulates morphogenetic processes in the mammary gland; and when hyper-expressed in transgenic mice, Notch-3 also promotes mammary gland carcinogenesis. Here, we show that Notch-3-dependent ERK activation in breast cancer via IL-6 targets the activation of *Jagged-1*, which belongs to a family of Notch ligands and *CA-IX*, a hypoxia survival gene. Consequently, our data suggest that IL-6 may trigger a potential autocrine/paracrine Notch-3/Jagged-1 loop to boost stem/progenitor self-renewal in the mammary gland. Finally, we present data regarding the autocrine IL-6 loop in breast cancer cells. In particular, we show that IL-6 upregulates its own mRNA, thus perpetuating the effects of transient IL-6 exposure of breast cancer cells. In addition, this autocrine IL-6 loop requires active Notch-3 expression. Hence, our data suggest that the upregulation of IL-6 gene expression in response to stress conditions (hypoxia) or to inflammation (IL-6 itself) may be maintained by an autocrine mechanism in Notch-3 stem/progenitor cells of the mammary gland.

## Abbreviations

Anti/α-IL-6: anti-IL-6 antibody
Anti/α-Notch-3: anti-Notch-3 antibody
CA-IX: carbonic anhydrase IX
GP96: heat shock protein GP96
GPR30: G-protein coupled receptor 30
Hypo-7 MCF-7: hypoxia derived subpopulation
IHC: immunohistochemical analysis
IL-6: interleukin-6
IL-6 Rα: IL-6 receptor
JAK: Janus kinase
MCF-7S: spheroids of MCF-7 cells
MMP2: metalloproteinase 2
MS: mammospheres
PIAS3: protein inhibitor of STAT3
pSTAT3: phosphorylated STAT3
STAT: signal transducer and activator of transcription
VEGF: vascular endothelial growth factor
WHO: World Health Organization
